# Clinical outcomes of patients with advanced hepatocellular carcinoma treated with sorafenib: a retrospective study of routine clinical practice in multi-institutions

**DOI:** 10.1186/s12885-015-1273-2

**Published:** 2015-04-08

**Authors:** Sae Hwan Lee, Il Han Song, Ran Noh, Ha Yan Kang, Suk Bae Kim, Soon Young Ko, Eoum Seok Lee, Seok Hyun Kim, Byung Seok Lee, An Na Kim, Hee Bok Chae, Hong Soo Kim, Tae Hee Lee, Young Woo Kang, Jae Dong Lee, Heon Young Lee

**Affiliations:** 1 Department of Internal Medicine, Dankook University College of Medicine, 201 Manghyang-ro, Dongnam-gu Cheonan, 330-715 Republic of Korea; 2 Department of Internal Medicine, Soonchunhyang University College of Medicine, Cheonan, Republic of Korea; 3 Department of Internal Medicine, Chungnam National University School of Medicine, Daejeon, Republic of Korea; 4 Department of Internal Medicine, Konyang University College of Medicine, Daejeon, Republic of Korea; 5 Department of Internal Medicine, Chungbuk National University College of Medicine, Cheongju, Republic of Korea; 6 Department of Internal Medicine, Eulji University College of Medicine, Daejeon, Republic of Korea; 7 Department of Internal Medicine, Konkuk University School of Medicine, Chungju, Republic of Korea

**Keywords:** Hepatocellular carcinoma, Cirrhosis, Sorafenib, Survival

## Abstract

**Background:**

Sorafenib is an orally administered multikinase inhibitor with antiangiogenic and antiproliferative properties. The results of large clinical trials demonstrate that sorafenib prolongs survival and the time to progression of patients with advanced hepatocellular carcinoma (HCC). The aim of the present study was to determine the outcomes of such patients who were routinely treated with sorafenib at multi-institutions in Korea, in contrast to formal clinical trials.

**Methods:**

Between August 2007 and March 2012, patients with advanced HCC in seven referral medical centers in Daejeon-Chungcheong Province of Korea were retrospectively enrolled to evaluate treatment response, survival, and tolerability following administration of sorafenib. The treatment response was assessed in accordance with the Response Evaluation Criteria in Solid Tumor 1.1 guidelines.

**Results:**

Among 116 patients, 66 (57%) had undergone treatment for HCC, and 77 (66%) were accompanied with Child-Pugh A cirrhosis. The median duration of sorafenib treatment was 67 days (range 14–452 days). Median overall survival and median time to progression were 141 days and 90 days, respectively. Complete response, partial response, and stable disease were achieved for 0%, 2%, and 29% of patients, respectively. Overall median survival, but not the median time to progression, was significantly shorter for patients with Child-Pugh B cirrhosis compared with those with Child-Pugh A cirrhosis (64 days vs 168 days, *P* = 0.004). Child-Pugh B cirrhosis (*P* = 0.024) and a high level of serum alpha-fetoprotein (*P* = 0.039) were independent risk factors for poor overall survival. Thirty-nine (34%) patients experienced grade 3/4 adverse events such as hand-foot skin reactions and diarrhea that required dose adjustment.

**Conclusions:**

The clinical outcomes of sorafenib-treated patients with advanced HCC were comparable to those reported by formal clinical trial conducted in the Asia-Pacific region. Underlying hepatic dysfunction was the most important risk factor for shorter survival.

## Background

Hepatocellular carcinoma (HCC) is the fifth most common malignancy worldwide and patient’s outcomes are generally poor [1,2]. Although potentially curable at an early stage, only 30-40% of patients with HCC at the time of diagnosis are eligible for curative treatments such as liver transplantation, tumor resection, or percutaneous radiofrequency ablation (RFA), and the majority of patients are obligated to undergo noncurative treatments such as transcatheter arterial chemoembolization (TACE) [3]. The role of systemic chemotherapy in treating patients with HCC at an advanced stage beyond these standard treatments was addressed by a few phase II/III clinical trials [4-6]. The poor outcomes of the majority of patients with HCC are presumably related to the aggressive behavior of HCC that causes progression to intrahepatic spread, invasion of large vessels, and distant metastasis.

Sorafenib is an orally administered drug that inhibits multiple protein kinases, including two transmembrane receptors as well as intracellular tyrosine and serine-threonine kinases, which together mediate tumor cell proliferation and angiogenesis [7,8]. During 2008–2009, the Sorafenib HCC Assessment Randomized Protocol (SHARP) and Asia-Pacific trials, which were international randomized controlled trials, showed a limited clinical benefit and acceptable safety profile of sorafenib treatment for patients with advanced HCC [9,10]. Although statistically significant, the increase in patients’ survival of approximately 2–3 months is disappointing to patients and their families. Further, radiological assessment revealed that the sum of complete and partial response rates was <5% in both trials. Unfortunately, few routine clinical data are available on the effects of sorafenib on patients with HCC in advanced stages with major vessel invasion or extrahepatic spread.

A greater knowledge of the efficacy and safety of sorafenib for treating patients with advanced HCC is required to develop treatment guidelines and to facilitate an informed decision-making process for patients with poor hepatic functional reserve or advanced liver cirrhosis [11,12]. The aims of the present study were to evaluate the treatment patterns, outcomes, and safety of sorafenib for routine treatment of Korean patients with advanced HCC with hepatic decompensation and compensated liver function.

## Methods

### Study population

We identified 146 patients with unresectable HCC who were treated with sorafenib between August 2007 and March 2012 in seven university referral hospitals located in Daejeon Metropolitan City and Chungcheong Province of Korea as follows: Dankook University Hospital, Soonchunhyang University Hospital, Chungnam National University Hospital, Konyang University Hospital, Chungbuk National University Hospital, Eulji University Hospital, and Konkuk University Hospital. The computerized medical records of these patients were retrospectively reviewed. After review, 30 patients were excluded as follows: 14 patients received <2 weeks of sorafenib treatment; seven patients with Child-Pugh C cirrhosis; five patients with no follow-up after the first visit for sorafenib treatment; three patients without adequate medical information, including the dose of sorafenib; and one patient without any evidence to support the diagnosis of HCC. The final 116 patients were assessed for treatment response, survival, and adverse effects. HCC was diagnosed according to either histologic or radiologic findings. Typical radiologic findings were as follows: a wash-out hepatic nodule on the portal or delayed hepatic venous phase following a hypervascular enhancing hepatic nodule on the arterial phase of imaging modalities such as dynamic computed tomography or magnetic resonance imaging with evidence of chronic liver disease regardless of the serum level of alpha-fetoprotein (AFP) [13]. HCC was considered unresectable according to the criteria as follows: extensive bilobar involvement of the liver due to single or multiple tumors; insufficient hepatic functional reserve (>15% of preoperative 15-min retention rate of indocyanine green); tumor invasion of major vessels such as portal or hepatic veins or the inferior vena cava; or an extrahepatic spread [14]. We conducted a review of patients’ medical records and collected data on the characteristics as follows: patient demographics, cause of liver disease, Eastern Cooperative Oncology Group (ECOG) performance status, hepatic functional reserve, previous treatments, and baseline tumor characteristics. The enrolled patients granted informed consent to evaluate their treatment of HCC. The Institutional Review Boards for Human Research of Dankook University Hospital, Soonchunhyang University Hospital, Chungnam National University Hospital, Konyang University Hospital, Chungbuk National University Hospital, Eulji University Hospital, and Konkuk University Hospital approved this study, which followed the ethical principles of the Declaration of Helsinki.

### Clinical outcomes and assessments

The primary endpoint of the study was overall survival, which was calculated from the date of sorafenib administration to the date of death from any cause. Secondary endpoints included the disease control rate with tumor response, time to radiological progression, and drug safety/tolerability. Tumor response was assessed according to the Response Evaluation Criteria in Solid Tumors (RECIST) version 1.1 [15]. The disease control rate was defined as the percentage of patients with the best rating of complete response, partial response, or stable disease according to RECIST criteria that was maintained for at least 4 weeks from the first manifestation of that rating. The time to radiological progression was defined as the time from sorafenib administration to tumor progression according to RECIST criteria. Drug safety and tolerability were classified in accordance with the National Cancer Institute Common Terminology Criteria for Adverse Events (NCI-CTCAE) version 3.0 [16].

### Statistical analysis

The data were expressed as the median (range) and the number (percentage). The overall survival and cumulative progression rates of HCC were evaluated using the Kaplan–Meier method with a log-rank test to determine the significance of the differences. Multivariate analysis performed to identify independent risk factors for overall survival using the Cox proportional hazard model after univariate analysis. *P* < 0.05 was considered statistically significant. All the analyses were performed using SPSS 14.0 software (SPSS Inc., Chicago, IL, USA).

## Results

### Patient characteristics

The baseline clinical characteristics of 116 patients with HCC who were treated with sorafenib are summarized in Table 1. The median age (range) of the patients was 56 (34–82) years, and 93 patients (80%) were male. Hepatitis B virus (HBV) was the most frequent cause of liver disease, followed in descending order by alcohol, hepatitis C virus (HCV), and others. Eighty-eight patients (76%) had an ECOG performance status score of 0 or 1, 77 patients (66%) had Child-Pugh A cirrhosis, and 93 patients (80%) had Barcelona Clinic Liver Cancer (BCLC) advanced-stage C. The proportion of patients with macrovascular invasion or extrahepatic metastasis was 55% or 46%, respectively. Sixty-six patients (57%) had undergone other treatments for HCC before receiving sorafenib, and the previous treatments and sessions are summarized in Table 2. TACE was the most frequent single treatment before administration of sorafenib. Surgical resection or RFA followed by TACE were the most frequent combined treatments. Radiological tumor progression before sorafenib treatment was identified in all study subjects, and the tumors of 34 patients (29%) were upstaged according to the modified International Union Against Cancer (UICC) staging system. The median follow-up period (range) after sorafenib treatment was 88 (21–1545) days.

**Table 1:** **Baseline characteristics of patients**

Characteristics	Subjects (n = 116)
Age, years*	56 (34–82)
Male gender, n (%)	93 (80)
Etiology, n (%)	
HBV/HCV/alcohol/others	79/6/18/13 (68/5/16/11)
Child-Pugh classification, n (%)	
A/B	77/39 (66/34)
ECOG performance status, n (%)	
0-1/2	88/28 (76/24)
History of prior treatment, n (%)	66 (57)
Modified UICC stage, n (%)	
II/III/IVa/IVb	2/26/30/58 (2/22/26/50)
BCLC stage, n (%)	
B/C/D	19/93/4(16/80/4)
AFP, ng/dL*	124 (1–150000)
Macrovascular invasion, n (%)	64 (55)
Extrahepatic metastasis, n (%)	54 (46)

*median (range).

*Abbreviations*: *HBV* hepatitis B virus, *HCV* hepatitis C virus, *ECOG* Eastern Cooperative Oncology Group, *UICC* International Union Against Cancer, *BCLC* Barcelona Clinic Liver Cancer, *AFP* alpha-fetoprotein.

**Table 2:** **Treatment modalities before sorafenib therapy**

Treatment modalities	Subjects (n = 66)
**Single treatment, n (%)**	**40 (61)**
TACE	35 (54)
Surgery	4 (6)
Radiation therapy	1 (1)
**Multidisciplinary treatment, n (%)**	**26 (39)**
Surgery followed by TACE	11 (17)
RFA followed by TACE	8 (12)
TACE and radiation therapy	4 (6)
RFA followed by TACE and radiation therapy	2 (3)
TACE followed by systemic chemotherapy	1 (1)
**Treatment session before sorafenib, n (%)**	
One	14 (21)
Two	10 (15)
Three or more	42 (64)

*Abbreviations*: *TACE* transcatheter arterial chemoembolization, *RFA* radiofrequency ablation.

### Treatment outcomes

**Figure 1:**
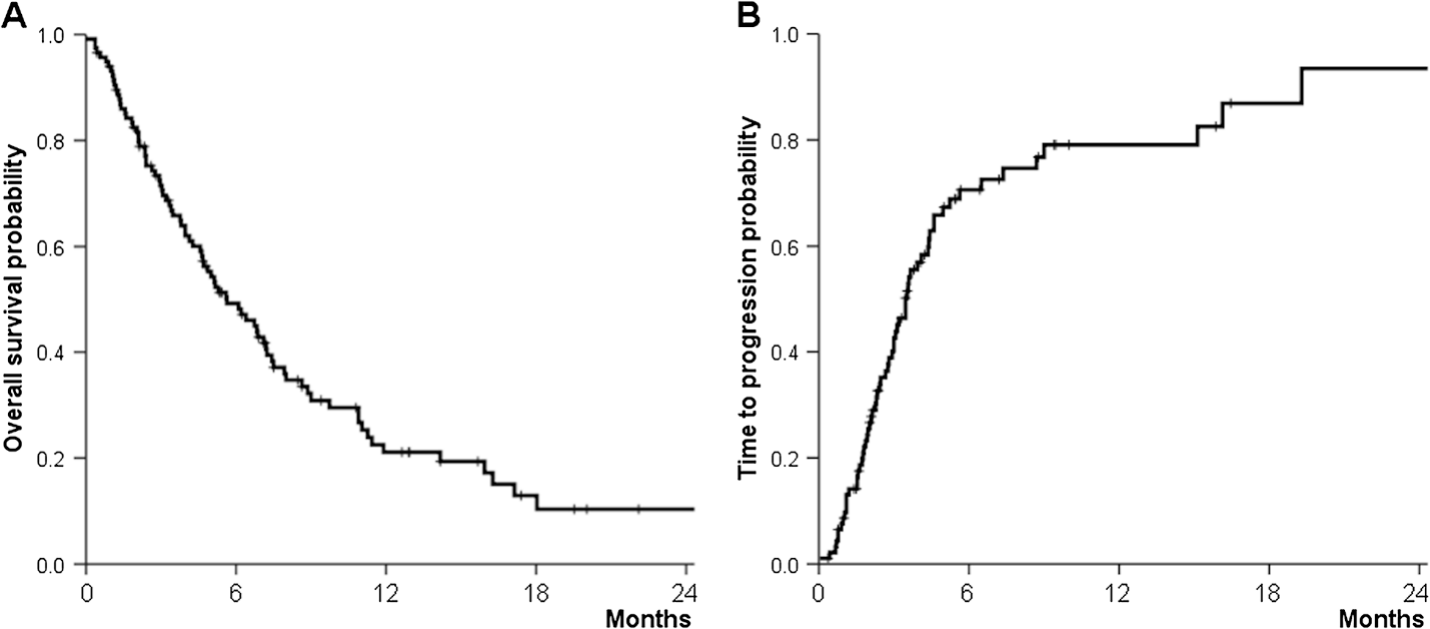
**Probability of overall survival (A) and the time to tumor progression (B).** The median overall survival was 141 days, and the survival rates at 6, 12, and 18 months were 47%, 21%, and 11%, respectively. The median time to tumor progression was 90 days.

**Figure 2:**
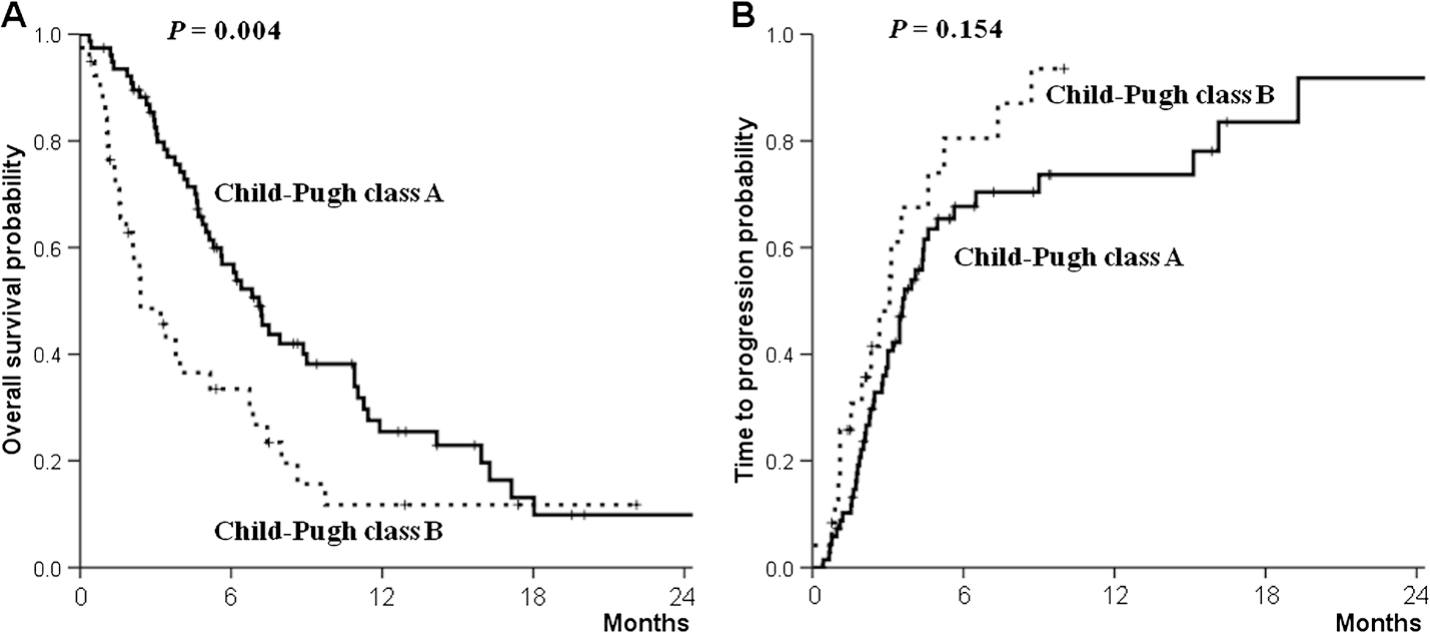
**Probability of overall survival (A) and the time to progression (B) according to Child-Pugh classification of cirrhosis.** The median overall survival of the HCC patients with Child-Pugh A cirrhosis was significantly longer compared with that of the HCC patients with Child-Pugh B cirrhosis (186 days vs 64 days, *P* = 0.004), although the median time to tumor progression did not differ between the two groups (104 days vs 63 days, *P* = 0.154).

Eighty-three (72%) patients died by the end of the follow-up period. Median overall survival was 141 (14–1581) days, and survival rates at 6, 12, and 18 months were 47%, 21%, and 11%, respectively (Figure 1A). The median follow-up period was 118 (28–1,581) days for 94 patients treated with sorafenib for at least 4 weeks who were administered a follow-up radiological evaluation and were available for assessment of their radiological response. The radiological responses of these patients were as follows (Table 3): 0 with a complete response; 2 (2%) with a partial response; 28 (29%) with stable disease; and 64 (69%) with progressive disease. The disease control rate was 21.9%. The median time to radiological progression of 64 patients with progressive disease was 90 (28–1,574) days (Figure 1B). The median overall survival of the HCC patients with Child-Pugh A cirrhosis was significantly longer compared with that of HCC patients with Child-Pugh B cirrhosis (186 vs 64 days, *P* = 0.004) (Figure 2A), although the median time to tumor progression did not differ between the groups (104 vs 63 days, *P* = 0.154) (Figure 2B).

**Table 3:** **Responses to sorafenib treatment**

	N = 94
Complete response	0 (0)
Partial response	2 (2)
Stable disease	28 (29)
Progressive disease	64 (69)
DCR (95% CI)	21.9% (23.7–42.8)

*Abbreviations*: *DCR* disease control rate, *CI* confidence interval.

### Prognostic factors for overall survival and tumor progression

Multivariate analysis revealed that Child-Pugh B cirrhosis (*P* = 0.024) and a serum level of AFP >200 ng/mL (*P* = 0.039) were independent prognostic factors for overall survival (Table 4). However, none of the clinical factors was associated with the time to tumor progression.

**Table 4:** **Univariate and multivariate analysis of risk factors for overall survival**

Factors	Univariate		Multivariate	
	OR (95% CI)*	*P*	OR (95% CI)*	*P*
Age (>60 years)	0.77 (0.49–1.22)	0.274		
Gender (male)	1.47 (0.81–2.67)	0.2	1.96 (0.96–4.01)	0.062
ECOG performance status (2)	1.13 (0.67–1.89)	0.629		
Etiology (HBV-related)	1.26 (0.79–1.98)	0.32		
Child-Pugh classification ( B)	1.93 (1.22–3.03)	0.004	1.84 (1.08–3.13)	0.024
History of prior treatment (yes)	0.68 (0.44–1.04)	0.08	0.78 (0.46–1.33)	0.376
AFP (>200 ng/mL)	1.63 (1.01–2.64)	0.047	1.76 (1.02–3.02)	0.039
Macrovascular invasion (yes)	1.56 (0.99–2.45)	0.054	1.96 (0.96–4.01)	0.062
Extrahepatic metastasis (yes)	1.21 (0.78–1.89)	0.383		

*odds ratio (95% confidence interval).

*Abbreviations*: *ECOG* Eastern Cooperative Oncology Group, *HBV* hepatitis B virus, *AFP* alpha-fetoprotein.

### Tolerability and safety of treatment

The median period of sorafenib treatment was 67 (14–452) days, and the median dose of sorafenib was 700 (105–800) mg per day. Table 5 shows drug-related adverse events and severe toxicity that each patient experienced predominantly according to liver function. Seventy-eight (67.2%) patients experienced drug-related adverse events such as hand-foot skin reaction (20%), diarrhea (16%), nausea (6%), anorexia (5%), fatigue (7%), or rash/desquamation (13%). Grade 3/4 toxicities occurred in 39 (33.6%) patients, which required a reduction in sorafenib dose to maintain treatment. However, the occurrence of grade 3/4 toxicities did not differ between patients with Child-Pugh A or B cirrhosis. Tumor progression was the most frequent cause of discontinuation of sorafenib treatment, followed by drug-related adverse events, hepatic failure, and an unacceptable economic burden, in decreasing order.

**Table 5:** **Incidence of drug-related adverse events according to liver function**

AE	All grades (n = 78)	Grade 3/4 (n = 39)		
		Child-Pugh A	Child-Pugh B	*P*
HFSR	23	10	4	0.340
Diarrhea	19	6	4	0.655
Nausea	7	1	0	0.475
Anorexia	6	4	1	0.510
Fatigue	8	2	1	0.991
Rash/desquamation	15	3	3	0.383

*Abbreviations*: *AE* adverse event, *HFSR* hand-foot skin reaction.

## Discussion

Until the advent of sorafenib, no systemic chemotherapy regimen improved the survival of patients with advanced HCC compared with those who received the best supportive care alone [17,18]. Although many clinical trials used several agents and combinations of these drugs, only sorafenib was approved by many countries for the treatment of patients with unresectable HCC, and sorafenib is now incorporated into practice guidelines [19-21]. Although sorafenib was the first agent to improve the overall survival of these patients, the therapeutic outcomes remain limited and unsatisfactory, even in well-designed randomized controlled trials. Therefore, This study was intended to assess the efficacy of sorafenib in actual clinical practice, in contrast to a clinical trial.

The median overall survival of approximately 4.7 months of the patients described here is inconsistent with the data of the SHARP [9] and Asia-Pacific trials [10]. When the present study commenced, we enrolled more patients with Child-Pugh B cirrhosis and ECOG performance status 2 compared with patients in those randomized trials. The different characteristics of patients indicate that the patients had decompensated hepatic reserve function and a poorer prognosis compared with those of the SHARP [9] or Asia-Pacific trials [10]. To reconcile our expectation with the findings of those trials, the survival rate of patients with Child-Pugh B cirrhosis was significantly lower compared with those diagnosed with Child-Pugh A cirrhosis. However, because it is uncertain whether the data may be explained by the effects of sorafenib or those of innate hepatic progressive disease, these results should be interpreted with caution.

The median time to progression of 3 months observed here was comparable with that reported by the Asia-Pacific trial [10] but not by the SHARP trial [9]. We believe that these results can be explained by an epidemiological similarity of the composition of the study populations enrolled in the present study and the Asia-Pacific trial [10], despite the difference in the hepatic reserve function of patients analyzed in each study. The distribution of endemic hepatitis virus differs among geographical regions. For example, 68% of the patients enrolled here had chronic HBV infection compared with 73% and 18% of those enrolled in the Asia-Pacific and SHARP trials, respectively. In contrast, the proportions of patients with chronic HCV infection recruited in our study and the Asia-Pacific trial were 5% and 8.4%, respectively, while that of the SHARP trial was 28%.

In contrast to the subanalysis results of overall survival, the time to tumor progression did not differ between patients with HCC with Child-Pugh A or B cirrhosis. This result indicates that the effect of hepatic functional reserve on tumor progression of patients treated with sorafenib may be insignificant. Although the disease control rate of our study was lower compared with those of the SHARP and Asia-Pacific trials, the result should be considered cautiously, because patients with HBV-related HCC survived for a significantly shorter time compared with those with HCV-associated HCC, and sorafenib may be less effective in HBV-positive patients with HCC [22]. Therefore, we suggest that a clinical assessment of sorafenib should be conducted more carefully for patients with advanced HCC in areas with endemic HBV infection.

Although sorafenib was originally approved as first-line treatment for unresectable HCC, it was administered as second-line treatment to more than half of the subjects in the present study after the tumor progression or recurrence following the initial treatments. In previous studies, prior treatment was associated with shorter overall survival rate but was not an independent risk factor for survival [23-25]. However, in the present study, baseline tumor characteristics and liver function were not significantly different between patients with or without prior treatment, and the overall survival of patients did not vary significantly depending on whether they received prior treatment. Taken together, these findings suggest that sorafenib is equally as effective as second-line treatment compared with its use as first-line treatment if patients have sufficient hepatic reserve. In the present study, Child-Pugh classification and serum AFP level, among several clinical parameters, were identified as independent predictors of overall survival. The results of other studies indicate that tumor characteristics (e.g. size, differentiation, vascular invasion, and extrahepatic metastasis) and patients’ performance status as well as liver function should be taken into account as prognostic determinants [26-28].

The frequency of drug-related adverse events noted in this study differed from those reported by other studies, although the types of adverse events were similar. In general, sorafenib-related adverse events occur more often in populations of Eastern countries compared with those of Western countries. The overall incidence of drug-related adverse events in this study was comparable to that of the Asia-Pacific trial, not the SHARP trial. The epidemiological differences associated with ethnicity may affect the development of drug-related adverse events, although this is unconfirmed. Grade 3/4 drug-related adverse events made it necessary to adjust the dose of sorafenib administered to 33.6% of patients in the present study. Hand-foot skin reactions and diarrhea were the major adverse events. However, similar to the results of other studies [23,25,29], these grade 3/4 toxicities did not differ with respect to reserve liver function.

Sorafenib was generally tolerated by patients with Child-Pugh class B disease, although their daily average dose of sorafenib was higher than that prescribed for patients with Child-Pugh class A. There was no significant difference in sorafenib tolerability or time to progression between the Child-Pugh class A and B groups, although overall survival was longer in the former group. In the present study, approximately two-thirds of the patients discontinued sorafenib treatment because of tumor progression or adverse events, each a key weakness of sorafenib treatment.

We note certain limitations of the present study. First, this was a retrospective study of patients’ medical records. Selection bias could not be avoided to some extent, and the information on drug compliance and adverse events may be insufficient. Next, this was a protocol-free, guideline-based study of routine clinical practice, in contrast to a formally designed and control study. Therefore, the possibility exists that the results were underestimated. Finally, this was not a comparative study. The lack of a control arm has the inherent limitation of analyzing clinical data independently from the results of prior studies.

## Conclusions

Here, we show that the outcomes of sorafenib treatment of Korean patients with advanced HCC in actual clinical practice was comparable to other studies conducted in the Asia-Pacific region in terms of tolerability and safety profiles. The median overall survival of patients with HCC with Child-Pugh A cirrhosis was significantly longer compared with that of HCC patients with Child-Pugh B cirrhosis, although the median time to progression did not differ between the groups. Further studies should be aimed to maximize the effect of sorafenib by selecting patients who will benefit the most.

## Abbreviations

HCC: hepatocellular carcinoma
RFA: radiofrequency ablation
TACE: transcatheter arterial chemoembolization
ECOG: Eastern Cooperative Oncology Group
RECIST: Response Evaluation Criteria in Solid Tumors
HBV: hepatitis B virus
HCV: hepatitis C virus
BCLC: Barcelona Clinic Liver Cancer
SHARP: Sorafenib Hepatocellular Carcinoma Assessment Randomized Protocol
AFP: alpha-fetoprotein
